# Opportunities and Challenges of Delivering Postabortion Care and Postpartum Family Planning During the COVID-19 Pandemic

**DOI:** 10.9745/GHSP-D-20-00263

**Published:** 2020-09-30

**Authors:** Anne Pfitzer, Eva Lathrop, Alison Bodenheimer, Saumya RamaRao, Megan Christofield, Patricia MacDonald, Bethany Arnold, Neeta Bhatnagar, Erin Mielke, Meridith Mikulich

**Affiliations:** a Jhpiego, Baltimore, MD, USA.; b Population Services International, Washington, DC, USA.; c United Nations Foundation, Washington, DC, USA.; d Population Council, New York, NY, USA.; e Bureau for Global Health, United States Agency for International Development, Washington, DC, USA.

## Abstract

Le tableau et encadre de dialogue de l’article est aussi disponible en français.

## Abstract (precis)

Devoting scarce health resources to meet the family planning needs of pregnant, postabortion, and postpartum women during the first wave of the COVID-19 pandemic is an investment against higher health systems burdens during subsequent waves of the pandemic and a means to save lives and improve livelihoods.

## Key Messages

We seek to assist decision makers in maximizing provision of essential services without compromising access to quality family planning care and while minimizing the risk of COVID-19 transmission among clients, and between clients and health care workers.

- Managers should help facility teams to integrate counseling and provide a range of contraceptive methods as is feasible within existing contacts with pregnant, postabortion, birthing, and postpartum women, even as services migrate to new models with a mixture of in-person and virtual/tele-health consultations.
- Policy makers should prioritize devoting resources to meet the family planning needs of pregnant, postabortion, birthing, and postpartum women, and the health care workers serving them as an investment against higher health systems burdens in later months and during subsequent waves of the pandemic.

## INTRODUCTION

The coronavirus disease (COVID-19) pandemic is poised to cause infection and death in millions of people across the globe at a stunning pace.^1^ The scale of the required response will inevitably pivot attention and resources toward fighting the pandemic and away from essential reproductive, maternal, newborn, and child health care, including access to voluntary family planning in the critical postabortion and postpartum periods. Decreased access to these lifesaving services will lead to a downstream increase in maternal and child morbidity and mortality.^2^^–^^4^ Data from previous complex emergencies demonstrate that a decrease in access to family planning results in increased poor outcomes related to unintended pregnancies and abortions.^5^ In a pandemic as vast and unique as COVID-19, where the primary mitigating factor is elimination of close physical contact, harnessing opportunities to provide family planning education, services, and supplies while women are already interfacing with the health care system during pregnancy and the postabortion, childbirth, and postpartum periods is strategic and lifesaving. This will require acceleration of integrated service delivery as well as creative and dynamic innovations of alternative service delivery approaches to address the family planning needs of pregnant, postabortion, birthing, and postpartum women. Investment in documentation of programmatic learnings could offer insights and opportunities for improving the resilience of health systems. Devoting scarce health resources to ensure the family planning needs of pregnant, postabortion, birthing, and postpartum women are met during the first wave of the COVID-19 pandemic is an investment against higher health systems burdens in later months and during subsequent waves of the pandemic and a means to ultimately save lives and improve livelihoods.

Harnessing opportunities to provide FP education, services, and supplies while women are already interfacing with the health care system is strategic and lifesaving.

## WHY FOCUS ON FAMILY PLANNING WITHIN SERVICES FOR PREGNANT, POSTABORTION, AND POSTPARTUM WOMEN NOW?

Closely spaced and unintended pregnancies are a public health concern and can have detrimental effects on women, infants, and children.^6^^–^^9^ The World Health Organization (WHO) recommends a 24-month interval from live birth until subsequent pregnancy to reduce the risk of adverse maternal, perinatal, and infant outcomes.^10^ Similarly, a woman who experiences a miscarriage or induced abortion that requires emergency treatment will rapidly return to fertility, with ovulation within 14–28 days depending on gestation.^11^ Thus, offering voluntary family planning counseling and services as part of postabortion care (PAC) as well as during pregnancy, childbirth, and the postpartum period is a critical means to protect vulnerable postpartum and postabortion women and reduce unintended and closely spaced pregnancies.^12^^,^^13^ Both postpartum and postabortion provision of voluntary contraceptive counseling and services constitute high-impact practices.^14^ In this commentary, we refer to the broad care of postabortion women as PAC, which includes^15^:

*two essential services: (1) treatment of emergency complications, and (2) voluntary family planning counseling, including provision of contraception.*

We use postabortion family planning (PAFP) when specifically referencing that component of PAC.

Before the COVID-19 pandemic, unmet need for modern contraception among women who wished to delay, space, or limit future childbearing and were not currently using a method in the postpartum and postabortion periods was already high.^16^^–^^18^ Because the COVID-19 pandemic has affected both supply- and demand-side access to family planning, women’s ability to achieve their reproductive intentions has been further compromised. Health systems worldwide seek to reduce facility visits to protect the health workforce and clients from the spread of COVID-19. Individuals’ health-seeking behavior is changing too, as they avoid facilities or seek care from alternate sources because of fear of acquiring the infection, respect for distancing measures, and/or mobility restrictions.

It is precisely here where postpartum family planning (PPFP) and PAFP offer a unique opportunity to make the most of facility and pharmacy visits and interactions with community health workers that individuals continue to have during the COVID-19 crisis. Now more than ever, and as others have already pointed out,^19^ the care that pregnant, postpartum, and postabortion women receive could be optimized to also meet their family planning needs by integrating contraceptive counseling and services for those who wish to space or limit their next pregnancy and to yield the significant health and well-being aims of voluntary contraceptive uptake and healthy timing and spacing of pregnancies.

PPFP and PAFP offer a unique opportunity to make the most of facility and pharmacy visits and interactions with community health workers that individuals continue to have during the COVID-19 crisis.

Thus, country health programs and facilities must prepare now and for the future to serve pregnant, postabortion, birthing, and postpartum women’s needs and to ensure women and their accompanying partners are informed, educated, and counseled on voluntary contraception. Although currently available guidelines center on infection prevention and control and immunization services, several international bodies have clarified the essential nature of family planning and maternal health care including the WHO (see these resources^20^ and these^21^).

## PRACTICAL APPROACHES AND MODIFIED FAMILY PLANNING SERVICE DELIVERY MODELS FOR DIVERSE POSTABORTION AND POSTPARTUM CLIENTS

To facilitate a reduction in COVID-19 transmission without compromising the quality of voluntary PAFP and PPFP counseling and services, we must enhance safe delivery of existing integrated service models (drawing lessons from previous emergencies) and also implement innovative, alternative service delivery mechanisms.

The key pillars in WHO’s strategy to reduce human-to-human transmission that must be incorporated into these models include:

1. Social distancing (e.g., supporting stay-at-home orders, limiting crowds in facilities, and reducing number of patient-provider contacts)
2. Early identification and isolation of cases (e.g., systematic screening, contact tracing, and community-based referral pathways)
3. Infection prevention and control (e.g., hand hygiene, appropriate personal protective equipment, and cleaning supplies)

We encourage countries to follow the WHO operational considerations^22^ for case management of COVID-19 and share WHO or locally adapted risk communication materials^23^ across all health sectors.

Health systems must confront rapidly changing challenges to maintain provision of essential health services, including PAC and PPFP. Overcoming these challenges will require a high level of intersectoral collaboration, communication, transparency, and community engagement. Specific obstacles will vary in number and magnitude by setting and may be particularly burdensome in already fragile settings. Early data highlight the high personal toll on health care workers (HCWs) during the pandemic in terms of their own physical health and risk of contracting the virus, mental health, structural concerns (availability/lack of personal protective equipment, long hours, etc.).^24^ Policy makers and program managers must identify which disruptions most significantly affect family planning outcomes so that limited resources can be allocated most effectively. These obstacles may include, but are not limited to:

- Supply chain disruptions for family planning and infection prevention and control products (e.g., stock-outs)^25^
- Clinic closures, reduced hours, and diminished capacity to treat high client volumes
- Redeployment of skilled family planning health staff to COVID-19 response
- HCW fear of contagion/contamination and attacks by community members
- Client hesitancy to access public transportation; health facilities treating COVID-19 patients and other crowded facilities (e.g., pharmacies, waiting rooms)
- Mobility or movement restrictions impacting clients and some cadres of the health workforce
- Income loss among individuals and families to access or pay user fees for contraceptive services
- Adoption of recommendations^26^ for limiting attendance of support people at in-person antenatal care (ANC), delivery, or postnatal visits; shifting of selected ANC visits to telehealth; and early postpartum discharge from facilities.

Policy makers and program managers must identify which disruptions most significantly affect family planning outcomes so that limited resources can be allocated most effectively.

On the last point, the recommendations inhibit or delay attributes of care in normal times, such as joint decision making around PPFP and may reduce time for PPFP counseling and method provision. Similar restrictions in PAC inhibit or delay male engagement in counseling and joint family planning decision making.

We highlight several approaches to address these challenges and maximize opportunities for voluntary PPFP and PAFP counseling and services (Table). Optimal voluntary adoption of PPFP and PAFP will be achieved not only through integration during provision of routine maternal and newborn care, but also by integrating family planning into other essential service contacts and outreach mechanisms.

**TABLE.** Recommendations Related to Integrating Postabortion and Postpartum Family Planning for Diverse Categories of Women^a^

Population	What PPFP or PAFP Service Is **Relevant** Here?	How Might This Service Be **Affected** by COVID-19 Mitigation?^b^	How Might You **Modify** the PPFP or PAFP Services in Context of COVID-19?	Key Resources
Women seeking ANC services	PPFP counseling at every ANC contact (whether in person or virtual) and messages reinforced at each subsequent contact to help women formulate a plan for voluntary PPFP.	ANC schedules and visits may be modified to allow for screening, triage, scheduling, social distancing, shifting some visits to virtual platforms or tele-consultations, providing ANC through CHWs, and/or by combining ANC contacts.	**All Women:** PPFP counseling at **each** of these ANC contacts remains paramount, particularly as disruptions to ANC may result in inconsistent models of care and providers throughout the pregnancy and beyond. Staff and clients follow local guidelines for wearing masks, especially when social distancing cannot be observed.	COVID-19 Technical Brief for Maternity Services (UNFPA)^26^ WHO recommendations on antenatal care for a positive pregnancy experience (includes PPFP ANC counseling)^27^ WHO Advice on the use of masks in the context of COVID-19^28^
			**Additional recommendations for women with positive COVID-19 test or symptoms**: As infrastructure allows and operating within local guidance, ensure that symptomatic women do not share the same space used by other women and that any shared spaces are cleaned between uses, offer telementoring of symptoms, and establish referral pathways for pregnant women requiring EmONC or treatment for severe COVID-19 disease.	
Women presenting for PAC	PAFP counseling to include all contraceptive methods and information on return to fertility. Voluntary FP service provision of all eligible methods.	Due to supply and/or service limitations, possible shifts to teleconsultations for medical management of abortion complications; the ability to provide a full range of FP options during PAC could be limited.	**All Women:** Where provision of a woman’s desired contraceptive method is not immediately possible, counsel about alternatives and plan for obtaining their preferred method once services and supply stabilize. When providing methods, observe all infection prevention and control protocols. For long-acting reversible methods, ensure client has a plan for managing side effects and for obtaining removal services. For short-acting methods, provide advance prescriptions and refills for several months depending on stock availability.	Infection prevention and control during health care when novel coronavirus (nCoV) infection is suspected^29^ Family Planning: A Global Handbook for Providers (2018 Update)^11^ A guide to preventing and addressing social stigma associated with COVID-19^30^
			**Additional for women with positive COVID-19 test or symptoms**: Place FP products (including self-administered methods and ECPs) at pharmacies for caregivers or trusted relatives of clients to access on their behalf and provide telehealth support.	
Women seeking maternity services	PPFP counseling to include all contraceptive methods and information on return to fertility, relative to exclusive breast-feeding practices. Voluntary service provision of all eligible methods.	After uncomplicated vaginal delivery, and where the home setting is suitable for recovery, health systems may consider early discharge with frequent telehealth monitoring for routine postnatal care and referrals for postpartum or newborn emergency care.	**All Women:** In these instances, continue offering PPFP counseling prior to discharge, emphasizing return to fertility and how and where to access PPFP during later postnatal or immunization visits. When providing FP methods, observe all infection prevention and control protocols. For long-acting reversible methods, ensure client has a plan for managing side effects and for obtaining removal services. For short-acting methods, consider advance dispensing ECPs and/or condoms for LAM users, or POPs or progesterone vaginal rings for those who will breastfeed, but not exclusively.	COVID-19 Technical Brief for Maternity Services (UNFPA)^26^ Advice on the use of point-of-care immunodiagnostic tests for COVID-19: scientific brief^31^
			**Additional recommendations for women with positive COVID-19 test or symptoms**: Ensure respectful care and companionship even if a woman delivers in area separate from asymptomatic women. Initiate breastfeeding promptly, with COVID-19 specific protective equipment for the mother. Plan for COVID-19related support post-discharge.	
Woman presenting for postnatal care and infant immunization services in facilities	PPFP counseling at PNC and immunization contacts. Voluntary FP service provision of all eligible methods.	Return postnatal care visits had low coverage in normal times and may be further compromised by COVID-19. Immunization coverage, while typically higher in normal times, is also affected by COVID-19.	**All Women:** Strengthen counseling during return postnatal care (PNC), and where appropriate, within immunization services. If privacy does not allow FP counseling during immunization/well baby clinic and staffing adequate, provide intra-facility linkage for FP for the mother. Consider utilizing lay counselors where clinical staff are overburdened or there are no staff providing FP services concurrently to immunization. In group education sessions, keep clients seated 2 meters apart; staff and clients follow local guidelines for wearing masks.	Guiding principles for immunization activities during the COVID-19^32^ Family Planning and Immunization Integration^33^
			**Additional for women with positive COVID-19 test or symptoms**: Same as with ANC, maintain adequate separation from asymptomatic women. Provide or reiterate mother with COVID-19 specific guidance for safe breastfeeding. Plan for additional COVID-19 related support.	
Pregnant, delivering and postpartum women not accessing facility-based services	PPFP and PAFP counseling at every community-based contact (whether in person or virtual), information on return to fertility, support for exclusive breastfeeding practices where applicable. Voluntary FP service provision and/or referral of all eligible methods.	Community- and home-based services may be utilized in greater magnitude due to limitations on facility-based care.	**All Women:** Emphasize PPFP and PAFP counseling and information on how to access services as part of CHW-led and other community-based initiatives, observing privacy and confidentiality. Health education platforms can also enhance education on PPFP and PAFP and educate women on how to use fertility awareness (with caution for women who have yet to see regular menstrual cycles return) and self-administered methods, such as LAM, POPs, and where available, progesterone vaginal rings. Various community stakeholders (women’s groups, community leaders, faith community) can also encourage essential services including PAC and PPFP, and provide support for finding these services. For in-person community-based care of pregnant, delivering, and postpartum women, services should be modified per local and international guidance to ensure safety, continuity, and protection of individuals and health workers. To provide FP methods, consider doorstep delivery of contraceptives, placing FP products at pharmacies, use of mobile outreach, deployment of digital applications that support self-administered and fertility awareness method use, referral to safe facility-based care where applicable. Where medical records allow, health workers may consider contacting women via phone to offer telehealth pregnancy and postnatal care, and to schedule safe facility-based visits.	Risk Communication and Community Engagement (RCCE) Action Plan Guidance COVID-19 Preparedness and Response^34^ Community-based health care, including outreach and campaigns, in the context of the COVID-19 pandemic^35^
			**Additional for women with positive COVID-19 test or symptoms**: Support through hotlines (or other remote means) for monitoring severity of symptoms and need for specialized care. Women with symptoms should be advised to stay away from community health events.		

^a^**General principles for all women:**
Promote respectful, stigma-free care, with cautious communication to blame the virus, not the person. This applies to women experiencing abortion complications as well.^30^ Promote task-sharing where CHWs already exist, including to encourage referrals to facility services and build trust through risk communication and community engagement. In the immediate postpartum or postabortion periods, women are, by default, nonusers after pregnancy. After method adoption, women may also need support to manage side effects and/or removal. Refer to contraception and COVID-19 guidance in such cases.^21^ Be on the lookout for signs of gender-based violence and support victims, as incidence of violence is expected to increase.^36^ Post or adapt WHO infographics on gender-based violence for displaying in health facilities and consider providing training to health workers who work with women on the 2016 WHO ANC recommendation which suggest clinical inquiry and referrals for GBV.^b^ **Factors that may influence implementation of PAC and PPFP services during the COVID-19 pandemic:** COVID-19 testing availability Country-specific laws, clinical guidelines and practice standards Task-sharing practices Pervasiveness of mobile phones and other communication technologies to facilitate “telehealth” Availability of IPC supplies (hand hygiene resources, personal protective equipment including masks for all HCWs and clients), environmental cleaning, and waste management) Stay-at-home orders and/or curfews Modifications to ANC, PAC, childbirth, PNC and immunization services Religious or cultural practices

Abbreviations: ANC, antenatal care; CHWs, community health workers; ECPs, emergency contraceptive pills; EmONC, emergency obstetric and newborn care; FP, family planning; GBV, gender-based violence; HCW, health care workers; LAM, Lactational Amenorrhea Method; LARC, long-acting reversible contraception; PAC, postabortion care; PAFP, postabortion family planning; PPFP, postpartum family planning; PNC, postnatal care; POPs, progestin-only pills; Q&A, question and answer; RH, reproductive health; UNFPA, United Nations Population Fund; WHO, World Health Organization.

These recommendations, of course, must be tailored to each unique setting for both logistical and cultural purposes. We also recognize that HCWs, especially in fragile settings, face myriad challenges during normal times which are only exacerbated during this pandemic. We hope HCWs recognize that maximizing opportunities with a client reduces the need for return visits and consequently the risks to themselves and their peers. Also, it enhances care for their clients in that it reduces the need to expose themselves to additional risks associated with separate family planning visits. Adjustments are required not only within health facilities, but throughout the health systems.

Maximizing opportunities with a client reduces the need for return visits and consequently the risks to clients, HCWs, and their peers.

We hope that program managers use the recommendations (Table) to promote these efforts in their communications with HCWs. This will encourage both HCWs and clients to feel safer, regardless of where they are seeking family planning information, products, and services. Thus, a blend of facility-based, community-based, and virtual/telehealth services could be used per setting, as context, health system, and community capacity allow. Additionally, providing clients with timely and accurate anticipatory guidance regarding changes to routine health care services will be essential in supporting their continued access to family planning. We encourage systems to monitor trends in utilization of various services along the continuum of care, at multiple levels, from facility to district to regional to national. We also encourage program managers to recognize HCWs and health facility teams who problem solve and innovate to optimize integration of services and suggest they document and disseminate process improvements and modifications so as to encourage appropriate replication.

## LEARNING RELATED TO PAC AND PPFP DURING COVID-19

Providers, policy makers, and those in positions of leadership can rely to some extent on past experiences in complex emergencies such as Ebola, Zika, and humanitarian responses to guide practice and service delivery in the context of the COVID-19 pandemic, but there remains much we do not know. Unique features of this disease can influence care differently than outbreaks of the past. For example, little is known about the impact of COVID-19 on pregnancy and postpartum recovery or the ways women’s and families’ health-seeking behaviors may change in the face of this pandemic. All provider cadres will likely experience a tension between duty of care and self-protection, and what educational messages, training, and protection strategies will work for them remains unknown. Others have highlighted that shifts toward self-care or short-acting contraceptive methods may have ripple effects, hopefully temporary, on the global supply chain for contraception^37^ that merit close monitoring. It is an imperative of the response community to explore gaps in our knowledge on both the health system and user sides, develop research protocols to generate answers, and document learning to inform ongoing care as COVID-19 continues to be a part of the new global reality (Box).

BOX. Knowledge Gaps in Postabortion Care and Postpartum Family Planning in COVID-19

**General Questions**

- What are the long-term impacts of COVID-19 on pregnancy/pregnancy loss?
- What is the extent to which COVID-19 affected equity of access to postabortion care (PAC) and postpartum family planning (PPFP) for marginalized and underserved women?
- What are the unique clinical feature of COVID-19 that may impact clinical care protocols?
- What contraceptive methods are women choosing during the pandemic (e.g., short-acting, long-acting, sporadic with emergency contraceptive pills)?
- How are health-seeking behaviors related to maternal and newborn health and family planning care changing in the context of COVID-19? And are there implications/differences in outcomes due to changes in behavior?
- Does stigma play a role in health-seeking behavior and decision making?
- What opportunities may exist for intersectoral coordination and linkages across public and private sectors within health and with non-health sectors COVID-19 response efforts (e.g., food distribution)?

**Questions Specific to PAC and PPFP**

- How do we communicate to communities about PAC and PPFP during pandemics?
- What concerns have women expressed about breastfeeding (as this may affect use of Lactational Amenorrhea Method)?
- What perceptions do providers have of caring for women seeking PAC, women in labor, and for women receiving procedures for long-acting reversible contraceptive methods in the context of COVID-19?
- What are the opportunities for task shifting these services?
- What policy changes were made due to COVID-19 to facilitate access to PAC and PPFP? Are these policies temporary or permanent?

## LINKAGES BETWEEN RESPONSE ON PAC AND PPFP AND HEALTH SYSTEM RESILIENCE

The emergence of COVID-19 has tested health systems worldwide, both in their management and mitigation of the pandemic directly, but also in their ability to maintain essential services for their populations. The WHO notes in the COVID-19 Operational Guidance for Maintaining Essential Health Services^38^:

*a system’s ability to maintain delivery of essential health services will depend on its baseline capacity and burden of disease*

alongside their COVID-19 transmission context. Thus, it is health systems’ **resilience**—or their capacity to prepare for and effectively respond to crises, maintain core functions when a crisis hits,^39^ and adapt and transform to function effectively post-pandemic^40^ —that offers a route to stymie COVID-19’s deleterious effects on essential health services both now and in subsequent waves of the pandemic.

A health system’s resilience could stymie COVID-19’s deleterious effects on essential health services now and in the future.

It is impossible to ignore the threats of not taking action. Based on experience from previous epidemics and health system shocks, we recognize that both family planning and maternal, newborn, and child health (MNCH) care and outcomes also stand to lose ground. One analysis of maternal and reproductive health outcomes estimates that a 10% decline in the use of essential care will result in 1.7 million additional women and 2.6 million additional newborns who will experience major complications as a direct result of care disruptions.^41^ Further, a 10% decline in modern contraceptive use would result in nearly 50 million additional women with unmet need for contraception.^41^ Amidst the Ebola outbreak in West Africa in 2014, maternal health stakeholders saw their coverage of ANC, facility delivery, and PNC drop.^42^ As health seeking patterns amidst COVID-19 appear to echo those witnessed during Ebola, experts estimate a similar, yet more substantial loss now—one which results in hundreds of thousands of additional child and maternal deaths.^43^

Amidst this gloomy outlook, MNCH programs are managing to provide services because pregnant women still need them. The ability to deliver these services comes in part due to rapid adaptations to provide safe care at community and household levels (including through self-care). Now, more than ever, the clarion call for integration of family planning with essential MNCH care appears: in the context of limited health service accessibility, optimizing every contact to uphold the health, well-being, and interests of women for their health and the health of their families. Simply put, PPFP and PAC integrate services to respond to individuals’ multidimensional needs with an array of simultaneous health interventions (in this case, voluntary family planning linked with maternal and/or infant health care). The health system adaptations we seek now and as we look to the future are both reactive to the moment we live in and an investment in the resilience of the system for the future. Opportunities for integration are central to—and should be capitalized upon—even in the midst of a crisis.

Amidst the gloomy outlook of COVID-19, MNCH programs are managing to provide services because pregnant women still need them.

## CONCLUSION

The ability of women, girls, and couples to freely choose the number, timing, and spacing of their pregnancies is a fundamental right and a means to achieve multiple sustainable development goals.^44^ Global actors have called for family planning to remain on the list of essential services during the COVID-19 pandemic, along with other key maternal, newborn, and child health care services.^45^^,^^46^ PAC and postpartum family planning intersect multiple categories of essential services. Prioritizing integrated service provision now promises to reap returns for improved health and well-being by preventing a rise in closely spaced pregnancies that may require care and burden facilities during subsequent waves of the epidemic. In the months to come, we can cultivate health system resilience by incorporating innovative models of integrated service delivery for pregnant, postabortion, delivering, and postpartum women; securing resources for programs to innovate and sustain services; and seeking partnerships between communities and MNCH programs and across the public and private sectors.

## Supplementary Material

20-00263-Supplement.pdf

## Notes

**Peer Reviewed**

**First published online:** September 17, 2020
