# Supplementary material for: Opportunities and Challenges of Delivering Postabortion Care and Postpartum Family Planning During the COVID-19 Pandemic
Source: Glob Health Sci Pract. 2020 Sep 30;8(3):335–43. doi: 10.9745/GHSP-D-20-00263 (PMC7541126; doi:10.9745/GHSP-D-20-00263)
Supplement: 20-00263-Supplement.pdf [file 20-00263-Supplement.pdf]

Supplement to: Pfitzer A, Lathrop E, Bodenheimer A, et al. Opportunities and challenges of delivering postabortion care and postpartum family planning during the COVID-19 pandemic. *Glob Health Sci Pract*. 2020;8(3). https://doi.org/10.9745/GHSP-D-20-00263

Tableau. Recommandations relatives à l'intégration de la planification familiale après avortement et post-partum pour diverses catégories de femmes

Population	Quel service de PFPA ou de PFPP est **pertinent** ici ?	Comment ce service pourrait-il **atténuer** l'impact de la COVID-19 ?	Comment pourriez-vous **modifier** les services de PFPA ou de PFPP dans le contexte de COVID-19 ?	Ressources clés
Femmes recherchant des soins prénataux	Conseils sur la PFPP à chaque contact prénatal (que ce soit en personne ou virtuellement) et messages renforcés à chaque contact ultérieur pour aider les femmes à formuler un plan de PFPP volontaire.	Le calendrier et les CPN peuvent être modifiés pour permettre le dépistage, le triage, la planification, la distanciation sociale, le déplacement de certaines visites vers des plateformes virtuelles ou des téléconsultations, la fourniture de CPN par l'intermédiaire des ASC, et / ou en combinant les contacts prénatals.	**Pour toutes les femmes :** Le conseil en PFPP **à chaque** contact prénatal reste primordial, d'autant plus que les perturbations des CPN peuvent entraîner des modèles de soins et de prestataires incohérents tout au long de la grossesse et au-delà. Le personnel et les clientes suivent les directives locales concernant le port des masques, en particulier lorsque la distanciation sociale ne peut être observée.<br><br>**Recommandations supplémentaires pour les femmes testées positives à la COVID-19 ou présentant des symptômes :** Dans la mesure où l'infrastructure le permet et fonctionne selon les directives locales, veiller à ce que les femmes symptomatiques ne partagent pas le même espace que les autres femmes et que tous les espaces partagés sont nettoyés entre les usages, proposer un télé mentorat des symptômes et établir des voies de référence pour les femmes enceintes nécessitant des SONU ou un traitement pour une maladie COVID-19 sévère.	COVID-19 Technical Brief for Maternity Services (Fiche technique COVID-19 pour les services de maternité, UNFPA)<br><br>Recommandations de l'OMS concernant les soins prénatals pour que la grossesse soit une expérience positive (y compris les conseils sur la PFPP pendant la CPN)<br><br>Conseils sur le port de masque dans le cadre de la COVID-19
Femmes se présentant pour des SAA	Les conseils en PFPA doivent inclure toutes les méthodes contraceptives et informations sur le retour à la fertilité. Prestation de services de PF volontaire pour toutes les méthodes éligibles.	En raison des limites de l'offre et / ou des services, il serait possible de passer aux téléconsultations pour la prise en charge médicale des complications de l'avortement ; la capacité à fournir une gamme complète d'options de PF pendant les SAA pourrait être limitée.	**Pour toutes les femmes :** Lorsque la fourniture de la méthode contraceptive souhaitée par une femme n'est pas immédiatement possible, il faut lui donner des conseils sur les alternatives et prévoir d'obtenir la méthode de son choix une fois que les services et l'offre se seront stabilisés.<br><br>Lors de la prestation de méthodes, respecter tous les protocoles de prévention et de contrôle des infections. Pour les méthodes réversibles de longue durée, s'assurer que la cliente dispose d'un plan pour la prise en charge des effets secondaires et pour obtenir des services de retrait. Pour les méthodes à court terme, prévoir des ordonnances à l'avance et des renouvellements pendant plusieurs mois en fonction de la disponibilité du stock.	Infection prevention and control during health care when novel coronavirus (nCoV) infection is suspected (Lutte anti-infectieuse lors de la prise en charge des patients chez nous sur suspecte une infection par un nouveau coronavirus (nCoV)

Supplement to: Pfitzer A, Lathrop E, Bodenheimer A, et al. Opportunities and challenges of delivering postabortion care and postpartum family planning during the COVID-19 pandemic. *Glob Health Sci Pract*. 2020;8(3). https://doi.org/10.9745/GHSP-D-20-00263

Population	Quel service de PFPA ou de PFPP est **pertinent** ici ?	Comment ce service pourrait-il **atténuer** l'impact de la COVID-19 ?	Comment pourriez-vous **modifier** les services de PFPA ou de PFPP dans le contexte de COVID-19 ?	Ressources clés
			**Recommandations supplémentaires pour les femmes testées positives à la COVID-19 ou présentant des symptômes** : Placer les produits de PF (y compris les méthodes auto-administrées et les PCU) dans les pharmacies pour que les soignants ou des membres de la famille en qui les clientes ont confiance puissent y accéder en leur nom, et fournir un soutien en télésanté.	Planification familiale : manuel a l'intention des prestataires de services du monde entier (mise à jour 2018)<br><br>Un guide pour prévenir et combattre la stigmatisation sociale associée au COVID-19
Femmes recherchant des services de maternité	Les conseils en PFPP doivent inclure toutes les méthodes contraceptives et informations sur le retour à la fertilité, relatives aux pratiques d'allaitement maternel exclusif. Prestation de services de PF volontaire pour toutes les méthodes éligibles.	Après un accouchement par voie basse sans complication, et lorsque le milieu familial est propice au rétablissement, les systèmes de santé peuvent envisager une sortie précoce avec un suivi fréquent par télésanté pour les soins postnatals de routine et des références pour des soins post-partum ou néonatals d'urgence.	**Pour toutes les femmes :** Dans ces cas, continuer à offrir des conseils sur la PFPP avant la sortie de l'établissement de santé, en mettant l'accent sur le retour à la fertilité et comment et où accéder aux services de PFPP lors des visites postnatales ou de vaccination ultérieure.<br><br>Lors de la prestation de méthodes de PF, observer tous les protocoles de prévention et de contrôle des infections. Pour les méthodes réversibles de longue durée, s'assurer que la cliente dispose d'un plan pour la prise en charge des effets secondaires et pour obtenir des services de retrait. Pour les méthodes à court terme, envisager la distribution anticipée de PCU et / ou de préservatifs pour les utilisatrices de la MAMA, ou des PPS ou l'anneau vaginal à la progestérone pour celles qui allaitent, mais pas exclusivement.	COVID-19 Technical Brief for Maternity Services (UNFPA)<br><br>Fiche technique COVID-19 pour les services de maternité (UNFPA)
			**Recommandations supplémentaires pour les femmes testées positives à la COVID-19 ou présentant des symptômes :** Assurer des soins respectueux et la présence d'un compagnon même si une femme accouche dans une zone distincte de celle des femmes asymptomatiques. Initier l'allaitement rapidement, avec un équipement de protection spécifique pour la COVID-19 pour la mère. Planifier le soutien lié à la COVID-19 suite au congé.	Advice on the use of point-of-care immunodiagnostic tests for COVID-19: scientific brief (Conseils sur l'utilisation de tests immunodiagnostiques au point de service

Population	Quel service de PFPA ou de PFPP est **pertinent** ici ?	Comment ce service pourrait-il **atténuer** l'impact de la COVID-19 ?	Comment pourriez-vous **modifier** les services de PFPA ou de PFPP dans le contexte de COVID-19 ?	Ressources clés
				pour le dépistage de la COVID-19)
Femme se présentant pour les soins postnatals et les services de vaccination infantile dans les établissements de santé	Conseils en PFPP lors des soins postnatals et contacts de vaccination. Prestation de services de PF volontaire pour toutes les méthodes éligibles.	Le suivi postnatal systématique a une faible couverture en temps normal et pourrait être compromis davantage par la COVID-19. La couverture vaccinale, bien que généralement plus élevée en temps normal, est également affectée par la COVID-19.	**Pour toutes les femmes :** Renforcer le conseil lors du suivi postnatal systématique et, le cas échéant, au sein des services de vaccination. Si le respect de la vie privée ne permet pas le conseil en PF pendant la vaccination / la clinique pour bébé en santé et si le personnel est suffisant, prévoir un lien entre les services de PF et la mère au sein de l'établissement de santé. Envisager de faire appel à des conseillers non professionnels lorsque le personnel clinique est surchargé ou qu'il n'y a pas de personnel fournissant des services de PF en même temps que la vaccination. Lors des séances d'éducation de groupe, s'assurer que les clientes sont assises à 2 mètres l'une de l'autre ; le personnel et les clientes suivent les directives locales en matière de port de masques.<br><br>**Recommandations supplémentaires pour les femmes testées positives à la COVID-19 ou présentant des symptômes** : Comme pour les CPN, maintenir une séparation adéquate des femmes asymptomatiques. Fournir ou réitérer à la mère des conseils spécifiques de COVID-19 pour un allaitement maternel sûr. Prévoir un soutien supplémentaire pour la COVID-19.	Principes directeurs relatifs aux activités de vaccination durant la pandémie de COVID-19<br><br>Intégration de la vaccination et de la planification familiale
Femmes enceintes, qui accouchent, et en post-partum n'accédant pas aux services dans les établissements de santé.	Conseils de PFPP et de PFPA à chaque contact communautaire (que ce soit en personne ou virtuel), informations sur le retour à la fertilité, soutien aux pratiques d'allaitement maternel exclusif le cas	Les services communautaires et à domicile peuvent être utilisés de manière plus importante en raison des limites des soins en établissement.	**Pour toutes les femmes :** Mettre l'accent sur les conseils et les informations sur la PFPP et la PFPA sur la façon d'accéder aux services dans le cadre d'initiatives menées par les ASC et d'autres initiatives communautaires, en respectant la vie privée et la confidentialité. Les plates-formes d'éducation sanitaire peuvent également améliorer l'éducation sur la PFPP et la PFPA et éduquer les femmes sur la manière d'utiliser la sensibilisation à la fertilité (avec prudence pour les femmes qui n'ont pas encore vu le retour de leurs cycles menstruels réguliers) et les méthodes auto-administrées, telles que la MAMA, les PPS, et le cas échéant, l'anneau vaginal à la	Communication sur les risques et participation communautaire (RCCE): Préparation et riposte face au nouveau coronavirus 2019<br><br>Soins de santé,

Supplement to: Pfitzer A, Lathrop E, Bodenheimer A, et al. Opportunities and challenges of delivering postabortion care and postpartum family planning during the COVID-19 pandemic. *Glob Health Sci Pract*. 2020;8(3). https://doi.org/10.9745/GHSP-D-20-00263

Population	Quel service de PFPA ou de PFPP est **pertinent** ici ?	Comment ce service pourrait-il **atténuer** l'impact de la COVID-19 ?	Comment pourriez-vous **modifier** les services de PFPA ou de PFPP dans le contexte de COVID-19 ?	Ressources clés
	échéant. Prestation de services de PF volontaire pour toutes les méthodes éligibles.		progestérone. Divers acteurs de la communauté (groupes de femmes, leaders communautaires, communauté religieuse) peuvent également encourager les services essentiels, y compris les SAA et la PFPP, et fournir un soutien pour trouver ces services.<br><br>Pour les soins communautaires en personne aux femmes enceintes, qui accouchent, et en post-partum, les services doivent être modifiés conformément aux directives locales et internationales afin d'assurer la sécurité, la continuité et la protection des personnes et des agents de santé.<br><br>Pour fournir des méthodes de PF, envisager la livraison à domicile de contraceptifs, le placement de produits de PF dans les pharmacies, l'utilisation de services mobiles de proximité, le déploiement d'applications numériques qui soutiennent l'auto administration et l'utilisation de méthodes de sensibilisation à la fertilité, la référence vers des soins sûrs en établissement de santé, le cas échéant.<br><br>Lorsque les dossiers médicaux le permettent, les agents de santé peuvent envisager de contacter les femmes par téléphone pour leur proposer des soins de grossesse et postnatals en télésanté, et pour planifier des visites sécurisées dans les établissements de santé.	activités de sensibilisation et campagnes communautaires dans le contexte de la pandémie de COVID-19
			**Recommandations supplémentaires pour les femmes testées positives à la COVID-19 ou présentant des symptômes** : Soutien via des lignes directes (ou d'autres moyens à distance) pour surveiller la gravité des symptômes et le besoin de soins spécialisés. Il faut conseiller aux femmes présentant des symptômes de ne pas assister à des événements de santé communautaire.	

Supplement to: Pfitzer A, Lathrop E, Bodenheimer A, et al. Opportunities and challenges of delivering postabortion care and postpartum family planning during the COVID-19 pandemic. *Glob Health Sci Pract*. 2020;8(3). https://doi.org/10.9745/GHSP-D-20-00263

<table>
<tr><th>Population</th><th>Quel service de PFPA ou de PFPP est pertinent ici ?</th><th>Comment ce service pourrait-il atténuer l’impact de la COVID-19 ?</th><th>Comment pourriez-vous modifier les services de PFPA ou de PFPP dans le contexte de COVID-19 ?</th><th>Ressources clés</th></tr>
<tr><td colspan="5">Principes généraux pour toutes les femmes :
● Promouvoir des soins respectueux et sans stigmatisation, avec une communication prudente pour blâmer le virus, pas la personne. Cela s'applique également aux femmes qui subissent des complications liées à l'avortement.
● Promouvoir le partage des tâches là où les ASC se trouvent déjà, notamment pour encourager la référence vers les services de l'établissement de santé et renforcer la confiance par la communication sur les risques et l'engagement communautaire.
● Dans les périodes post-partum ou après avortement immédiats, les femmes sont, par défaut, des non utilisatrices après la grossesse. Après l'adoption de la méthode, les femmes peuvent également avoir besoin de soutien pour la prise en charge des effets secondaires et / ou le retrait. Se référer aux directives relatives à la contraception et à la COVID-19 dans de tels cas.
● Savoir reconnaitre les signes de violence basée sur le genre et soutenir les victimes, car l'incidence de la violence devrait augmenter.
● Publier ou adapter des infographies de l'OMS sur la violence basée sur le genre pour les afficher dans les établissements de santé et envisager de former les agents de santé qui travaillent avec des femmes sur la recommandation 2016 de l'OMS sur les soins prénatals qui suggèrent une enquête clinique et des références pour la VBG.</td></tr>
<tr><td colspan="5">Facteurs pouvant influencer la mise en œuvre des services de SAA et de PFP pendant la pandémie de COVID-19 :
● Disponibilité des tests COVID-19
● Lois, directives cliniques et normes de pratique spécifiques à chaque pays
● Pratiques de partage des tâches
● L'omniprésence des téléphones mobiles et d'autres technologies de communication pour faciliter la « télésanté »
● Disponibilité des fournitures de PCI (ressources pour l'hygiène des mains, équipement de protection individuelle, y compris des masques pour tous les agents de santé et les clientes), nettoyage de l'environnement et gestion des déchets)
● Ordres de confinement et / ou couvre-feu
● Modifications des services de CPN, de SAA, d'accouchement, de soins postnatals et de vaccination
● Pratiques religieuses ou culturelles</td></tr>
</table>

Abréviations : CPN, soins prénatals ; ASC agents de santé communautaire ; PCU, pilules contraceptives d'urgence ; SONU, soins obstétriques et néonataux d'urgence ; PF, planification familiale ; VBG, violence basée sur le genre ; MAMA, méthode de l'allaitement maternel et de l'aménorrhée ; MLD, contraception réversible de longue durée ; SAA, soins post-avortement ; PFPA, planification familiale post-avortement ; PFPP, planification familiale du post-partum ; PPS, pilules a progestatif seul ; Q&R, questions-réponses ; SR, santé de la reproduction ; UNFPA, Fonds des Nations unies pour la population ; OMS, Organisation mondiale de la Santé ; PCI, prévention et contrôle des infections.

Supplement to: Pfitzer A, Lathrop E, Bodenheimer A, et al. Opportunities and challenges of delivering postabortion care and postpartum family planning during the COVID-19 pandemic. *Glob Health Sci Pract*. 2020;8(3). https://doi.org/10.9745/GHSP-D-20-00263

ENCADRÉ. Lacunes dans les connaissances sur les soins après avortement et la planification familiale en post-partum durant la COVID-19

Questions générales

- Quels sont les impacts à long terme de la COVID-19 sur la grossesse/les pertes de grossesse ?
- Dans quelle mesure, la COVID-19 a-t-elle affecté l'équité d'accès aux soins après avortement (SAA) et à planification familiale en post-partum (PFPP) pour les femmes marginalisées et mal desservies ?
- Quelles sont les caractéristiques cliniques uniques de la COVID-19 qui peuvent avoir un impact sur les protocoles de soins cliniques ?
- Quelles méthodes contraceptives les femmes choisissent-elles pendant la pandémie (par exemple, à courte, longue ou longue durée d'action, à action sporadique, etc. avec des pilules de contraception d'urgence) ?
- Comment les comportements liés à la recherche de soins de santé maternelle et néonatale et de planification familiale évoluent-ils dans le contexte de la COVID-19 ? Et y a-t-il des implications/différences dans les résultats en raison de changements de comportement ? La stigmatisation joue-t-elle un rôle dans les comportements de recherche de soins et la prise de décision ?
- Quelles sont les possibilités de coordination intersectorielle et de liens entre les secteurs public et privé dans le cadre de la santé et en dehors du secteur sanitaire quant aux efforts de riposte à la COVID-19 (par exemple, la distribution alimentaire) ?

Questions spécifiques aux SAA et à la PPFP

- Comment communiquer aux communautés concernant les SAA et la PPFP pendant les pandémies ?
- Quelles sont les préoccupations exprimées par les femmes au sujet de l'allaitement maternel (car cela peut affecter le recours à la méthode de l'allaitement maternel et aménorrhée - MAMA) ?
- Quelles sont les perceptions des prestataires quant à la prise en charge des femmes cherchant des SAA, ou femmes en travail ou pour les femmes recevant des procédures pour méthodes contraceptives à longue durée d'action dans le cadre de COVID-19 ?
- Quelles sont les possibilités de délégation des tâches pour ces services ?
- Quels changements de politique sont survenus à la suite de la COVID-19 pour faciliter l'accès aux SAA et à la PPFP ? Ces politiques sont-elles temporaires ou permanentes ?

Traduit avec www.DeepL.com/Translator (version gratuite) et puis, traduction revue et éditée par Anne Pfitzer
